# 2D NMR Methods for Structural Delineation of Copper(II) Complexes of Penicillin and Pilocarpine

**DOI:** 10.1155/MBD.1994.279

**Published:** 1994

**Authors:** Elena Gaggelli, Nicola Gaggelli, Gianni Valensin

**Affiliations:** 1 Department of Chemistry University of Siena Pian dei Mantellini 44 Siena I-53100 Italy

## Abstract

A method was developed for delineating the structure of paramagnetic metal complexes. The selective disappearance of cross-peaks in proton-carbon shift correlated 2D NMR maps was shown to uniquely depend upon the scalar and/or dipolar interaction between ligand nuclei and the unpaired electron(s), thus providing a means of identifying binding sites. Copper(II) was shown to form metal complexes with both Penicillin (PNC) and Pilocarpine (PLC) and the structure of the two 1:2 complexes in water solution at physiological pH were determined.


### 2D NMR METHODS FOR STRUCTURAL DELINEATION OF COPPER(II) COMPLEXES OF PENICILLIN AND PILOCARPINE

Elena Gaggelli, Nicola Gaggelli and Gianni Valensin*

Department of Chemistry, University of Siena, Pian dei Mantellini 44, I-53100 Siena, Italy

### Abstract

A method was developed for delineating the structure of paramagnetic metal complexes. The selective disappearance of cross-peaks in proton-carbon shift correlated 2D NMR maps was shown to uniquely depend upon the scalar and/or dipolar interaction between ligand nuclei and the unpaired electron(s), thus providing a means of identifying binding sites. Copper(II) was shown to form metal complexes with both Penicillin (PNC) and Pilocarpine (PLC) and the structure of the two 1:2 complexes in water solution at physiological pH were determined.

---

### INTRODUCTION

Metal ions are being given growing interest by the pharmacologist: not only several metallorganic and metalloinorganic molecules have been shown to possess wide ranges of biopharmaceutical activities, but also the effects of several drugs have been demonstrated to be modulated by the presence of selected metal ions.

However, the following problem has received poor, if any, attention. Most drugs are flexible ligands that, in solution, give rise to conformational equilibria among several arrangments of similar conformational energy; it is on the contrary agreed that the drug bound to its receptor assumes a unique conformation that does not necessarily corresponds to the solid state structure. Thus binding to the receptor and, hence, eliciting the biological action also depends on a process of conformational selection that is likely to involve a role of the ubiquitous metal ions, such as calcium, zinc, copper etc. Comprehension of metal-ligand equilibria therefore plays a major role in determining the base for a rationale drug design.

NMR methods provide a great piece of information on metal-ligand structures and equilibria, especially when the metal ion is a paramagnetic one. In such a case the up- or down-field shifts of nuclear resonances or the large enhancements of nuclear relaxation rates provide a unique tool for delineating the structure of metal complexes, as thoroughly reviewed elsewhere (1-4). The main drawback of these methods is that a well resolved NMR spectrum is required and also that the theory can be applied only in situations where the metal complex is much less abundant than the free ligand and in a restricted range of exchange rates (5). In fact structural information can only be gained when $R_{1b} >> k_b$, where $R_{1b}$ is the nuclear spin-lattice relaxation rate in the metal-complexed environment and $k_b$ is the kinetic constant for the ligand leaving the metal coordination sphere.

Here we present a novel NMR method that yields a direct structural elucidation of paramagnetic metal complexes by using common 2D techniques, such that resolution limits can be overcome. The structures of the complexes formed by copper(II) ions with pilocarpine, a cholinomimetic agent, and penicillin G are delineated as an example of the potential of the method.

### EXPERIMENTAL

Pilocarpine (PLC) {(3S-cis)-3-ethyldihydro-4-[(1-methyl-1H-imidazol-5-yl)methyl]-2(3H)-furanone, the chief alkaloid obtained from the leaves of South American shrubs of the genus *Pilocarpus*}

and penicillin G (PNC) were purchased from Sigma Chemical Co. and used without further purification. Solutions of PLC were made in deuterium oxide 99.95 % (Merck) and adjusted in pH with either DCl or NaOD. Solutions of PEN were made in [$^2H_6$]-dimethylsulfoxide 99.5 % (Merck). Both solutions were carefully deoxygenated by bubbling nitrogen gas. The desired concentration of copper ions in either deuterium oxide or [$^2H_6$]-dimethylsulfoxide solution was achieved by using stock solutions of copper nitrate. The exact concentration of copper in solution was determined by atomic absorption.

All NMR measurements were performed on a Varian VXR-200 spectrometer by using standard procedured for obtaining homo- and heteronuclear shift correlated 2D spectra (6,7). The intensity of diagonal and cross peaks in the 2D contour maps was measured by the integral of the peak as obtained by the 1D traces.

### THEORY

In order to ascertain the effect of paramagnetic ions on the appearance of 2D shift correlated contour maps, the simple $^{13}C$-$^{1}H$ AX spin system is considered. Under spin-decoupling conditions the hetero-COSY and the HOESY spectra both yield identical contour maps with a unique peak correlating the $^{13}C$ and the $^{1}H$ resonances. The difference in the two pulse sequences (the HOESY experiment has an additional 90° pulse and an additional mixing time) determines that scalar and dipolar connectivities are respectively shown in the COSY and HOESY spectra. The intensity of the cross-peak in the HOESY spectrum can be calculated by (8):

$$a_{CH}(t_m) = -\frac{M_0}{2}\frac{R_{CH}}{R_T}e^{-R_L t_m}\left\{1-e^{-R_T t_m}\right\} \qquad [1]$$

where $t_m$ is the mixing time, $M_0$ is the equilibrium longitudinal magnetization, $R_{CH}$ is the dipolar cross-relaxation rate, $R_T$ measures the rate of transfer between the two spins and $R_L$ determines the rate of leakage of magnetization towards the lattice, as given by the following equations (9):

$$R_{CH} = \frac{1}{10}\left(\frac{\gamma_C\gamma_H\hbar}{r_{CH}^3}\right)^3\left\{\frac{6\tau_c}{1+(\omega_H+\omega_C)^2\tau_c^2}-\frac{\tau_c}{1+(\omega_H-\omega_C)^2\tau_c^2}\right\}$$

$$R_T = \sqrt{(R_{1C} - R_{1H})^2 + 4R_{CH}}$$

$$R_L = \frac{1}{2}(R_{1C} + R_{1H}) - \frac{1}{2}R_T$$

where

$$R_{1C} = \frac{1}{10}\left(\frac{\gamma_C\gamma_H\hbar}{r_{CH}^3}\right)^3\left\{\frac{3\tau_c}{1+\omega_C^2\tau_c^2} + \frac{6\tau_c}{1+(\omega_H+\omega_C)^2\tau_c^2} + \frac{\tau_c}{1+(\omega_H-\omega_C)^2\tau_c^2}\right\} + R_{ext}^C$$

$$R_{1H} = \frac{1}{10}\left(\frac{\gamma_C\gamma_H\hbar}{r_{CH}^3}\right)^3\left\{\frac{3\tau_c}{1+\omega_H^2\tau_c^2} + \frac{6\tau_c}{1+(\omega_H+\omega_C)^2\tau_c^2} + \frac{\tau_c}{1+(\omega_H-\omega_C)^2\tau_c^2}\right\} + R_{ext}^H$$

In all the above equations $\omega$ reprsents the Larmor frequencies, $\gamma$ the magnetogyric ratios, $\tau_c$ the motional correlation time and $R_{ext}$ any contribution to the relaxation mechanism other than the dipolar one. By assuming a 100% dipolar contribution, $r_{CH}$ = 1.09 Å and a motional correlation time of 0.1 ns, the intensity of the cross-peak can be calculated as a function of $t_m$ at any value of the external magnetic field. For example at $B_o$ = 4.7 T the data shown in Table 1 is obtained.

**Table 1.** Intensity of the cross-peak in a HOESY spectrum as a function of the mixing time

| $a_{CH}(t_m)$ $(I/I_{max})$x100 | $t_m$ (ms) |
|---|---|
| 0.0 | 0 |
| 24.0 | 50 |
| 46.0 | 100 |
| 72.0 | 200 |
| 84.0 | 300 |
| 93.0 | 400 |
| 99.0 | 500 |
| 98.0 | 600 |
| 90.0 | 700 |

If the same spin system is considered in a paramagnetic metal complex, the occurrence of a strong source of "external" relaxation determines the fast disappearence of the cross peak at increasing concentration of the paramagnetic ion. In fact, as shown in Table 2, it can be easily calculated that the cross-peak almost disappears for an "external" contribution of *ca.* 20 $s^{-1}$.

The consequence of such effect can be evaluated by considering a copper(II) complex, where the paramagnetic ion is found at 4 Å from both the $^{13}C$ and the $^{1}H$ nuclei. Such assumption allows to calculate the contribution of the dipolar electron spin-nuclear spin interaction to both the $^{13}C$ and $^{1}H$ nuclear spin-lattice relaxation rates at 116 $s^{-1}$ and 1780 $s^{-1}$ respectively. The conclusion is that there is not any chance of detecting cross-peaks in the 2D maps obtained with a paramagnetic metal complex.

**Table 2.** Intensity of the cross-peak in a HOESY spectrum as a function of the "external contribution" to the spin-lattice relaxation rate

| $a_{CH}(t_m)$ $(I/I_{max})$x100 | $R_{ext}$ $(s^{-1})$ |
|---|---|
| 100.0 | 0 |
| 67.0 | 2 |
| 38.0 | 4 |
| 32.0 | 6 |
| 20.0 | 8 |
| 14.0 | 10 |
| 8.0 | 15 |
| 4.0 | 20 |
| 2.0 | 25 |

However in situations where the ligand can exchange between the free bulk state and the metal coordination sphere, being this last environment much less abundant than the other one, the intensity of the cross-peak is determined by the eigenvalues of either the spectral or the exchange matrices; the consequence is that closeness to the paramagnetic ion selectively affects the volume of correspondent cross peaks in COSY as well as in HOESY spectra (10).

### RESULTS AND DISCUSSION

The HOESY map obtained with pilocarpine 100 mM in deuterium oxide buffered at pH 5.5 and at T = 300 K is shown in Figure 1. Any cross-peak in the map represents a one-bond $^{13}C$-$^{1}H$ dipolar connectivity and belongs to the $^{13}C$-$^{1}H$ spin systems numbered as in the molecular formula. The addition of copper perchlorate gave rise to selective decrease of the cross-peaks' linewidths and intensities such that at [$Cu^{++}$] = 0.13 mM the cross-peaks boxed in Figure 1 had almost disappeared.

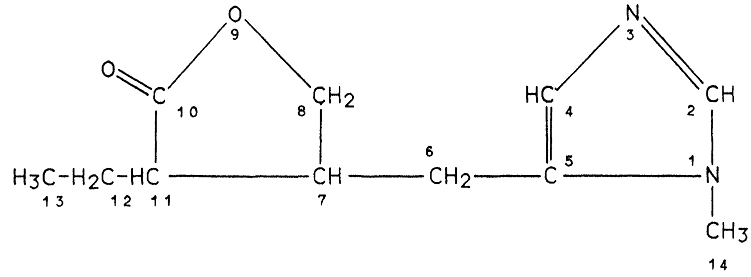

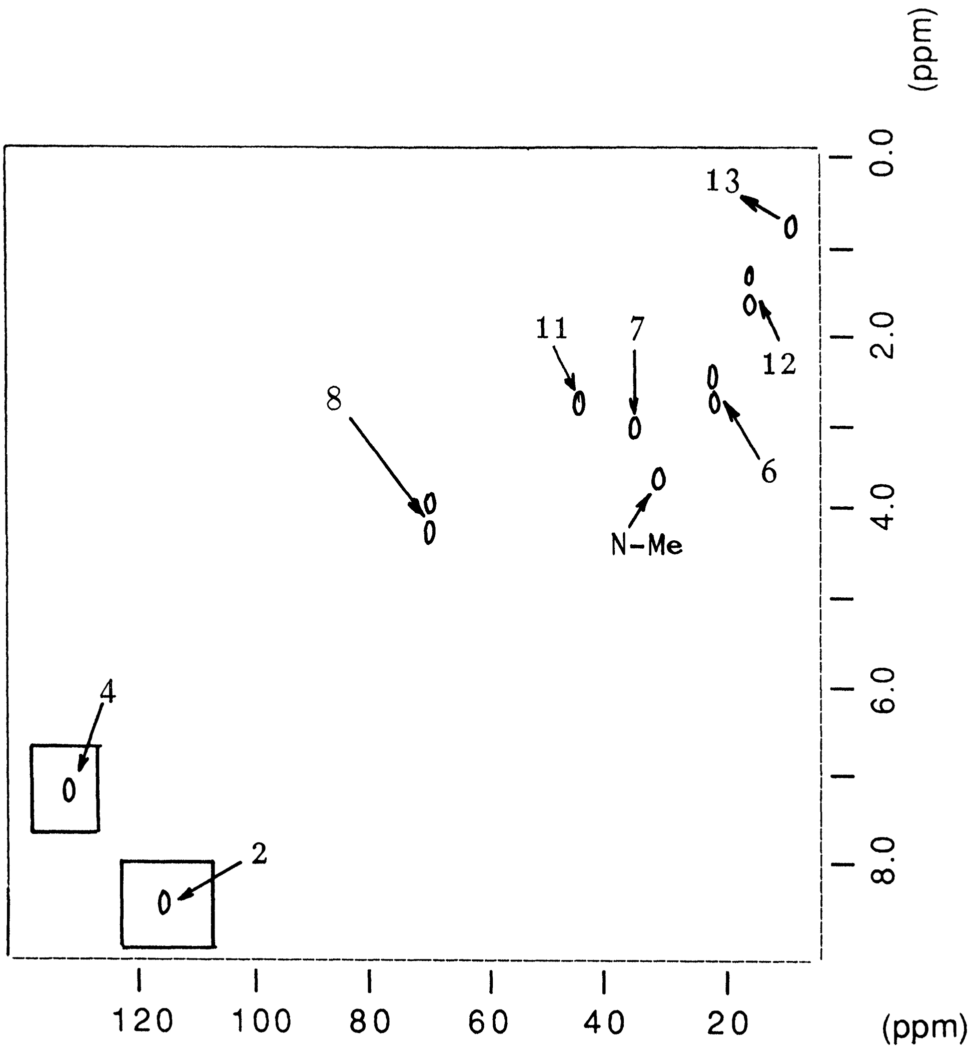

These data supports the evidence of an axclusive involvement of the pyridine-type imidazole nitrogen in metal-binding. Since pilocarpine hydrochloride was shown to preferentially occur in water solution in a conformation characterized by the absence of folding (11), the following conclusions could be drawn:

(i) copper binds to pilocarpine

(ii) binding of copper occurs at the imidazole protonation site

(iii) binding of copper does not result in any evident change of conformation of the ligand.

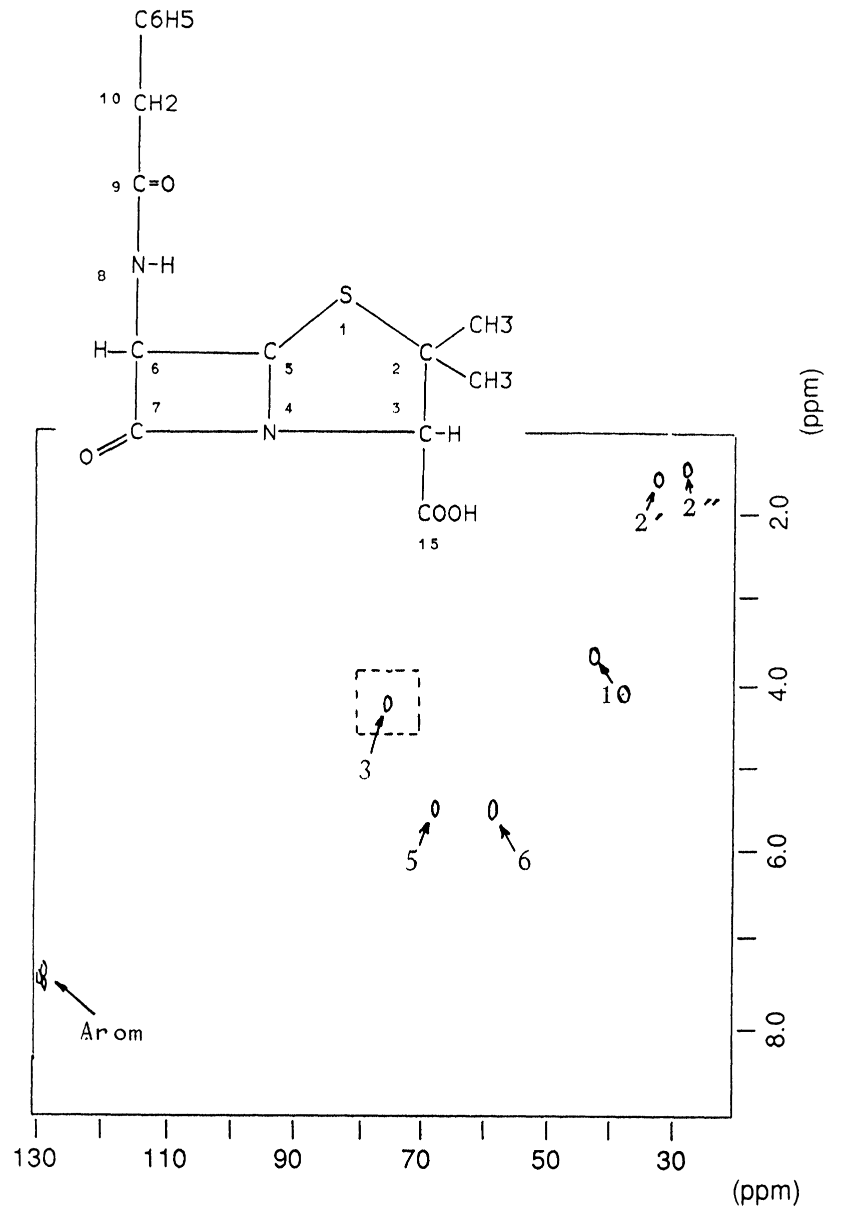

The hetero-COSY map obtained for penicillin G 10 mM in [$^2H_6$]-DMSO at T=300 K is shown in figure 2. In this case the cross-peaks represent the one bond $^{13}C$-$^1H$ scalar connectivities of the different $^{13}C$-$^1H$ spin systems. Addition of copper ions only affected the linewidths in a selective way such that at [$Cu^{2+}$]=0.022 mM the boxed cross-peak in the figure had been widened beyond detection limits. Consideration of the possible sites for metal complexation led to conclude that copper is bound to the carboxyl group and to the thiazolidine tertiary nitrogen. Any involvement of the side chain in metal binding was excluded by the absence of any paramagnetic effect upon the $C_6$-$H_6$ or $C_{10}$-$H_{10}$ spin systems.

The examples reported herein for very simple molecules should be taken as to demonstrate the potential of 2D NMR methods in delineating the structure of paramagnetic metal complexes. From this point of view, scalar or dipolar $^{13}C$-$^1H$ shift correlated 2D NMR contour maps offer similar opportunities of easy and direct detection of metal binding sites. At a deeper level of knowledge it must be however stressed that a comparison of paramagnetic effects on cross-peaks in hetero-COSY and HOESY maps even allows to discriminate between dipolar and scalar interactions with the unpaired electron spin (10).

**REFERENCES**

(1) R.A.Dwek, *Nuclear Magnetic Resonance in Biochemistry*, Clarendon Press, Oxford, UK, 1975.

(2) N.Niccolai, E.Tiezzi and G.Valensin, *Chem. Rev.* **82**, 359 (1982).

(3) I.Bertini and C.Luchinat, *NMR of Paramagnetic Species in Biological Systems*, Benjamin Cummings, Menlo Park, CA, 1986.

(4) G.Navon and G.Valensin, in *Metal Ions in Biological Systems*, H.Sigel ed., Marcel Dekker, New York, 1987, Vol.21, p. 1.

(5) T.J.Swift and R.E.Connick, *J.Chem.Phys.* **37**, 307 (1962).

(6) A.A.Maudsley and R.R.Ernst, *Chem.Phys.Letters* **50**, 368 (1977).

(7) P.L.Rinaldi, *J.Am.Chem.Soc.* **105**, 5167 (1983).

(8) J.Jeener, B.H.Meier, P.Bachmann and R.R.Ernst, *J.Chem.Phys.* **71**, 4546 (1979).

(9) S.Macura and R.R.Ernst, *Mol.Phys.* **41**, 95 (1979).

(10) E.Gaggelli, A.Maccotta and G.Valensin, *Inorg.Chem.* **32**, 2788 (1993).

(11) E.Gaggelli, N.Gaggelli, G.Valensin and A.Vivi, *Can.J.Chem.* **71**, 738 (1993).

**Received: November 30, 1993**

